# The Effects of Rhubarb for the Treatment of Diabetic Nephropathy in Animals: A Systematic Review and Meta-analysis

**DOI:** 10.3389/fphar.2021.602816

**Published:** 2021-06-11

**Authors:** Jing-Yi Zeng, Yu Wang, Miao Miao, Xiao-Rong Bao

**Affiliations:** Department of Nephrology, Jinshan Hospital, Fudan University, Shanghai, China

**Keywords:** rhubarb, rhein, emodin, diabetic nephropathy, animals, meta-analysis

## Abstract

**Background:** Rhubarb, also known as Da Huang, is a traditional Chinese medicine, and it was often used as a laxative in the past. Recently, multiple studies have applied rhubarb to treat diabetic nephropathy (DN). Anthraquinones, including emodin and rhein, have been extracted from rhubarb and used to explore the effective components and possible mechanisms of rhubarb for DN. Evaluating the efficacy of rhubarb may provide a scientific reference for the clinical application of rhubarb for the treatment of DN.

**Objective:** 1) To evaluate the efficacy of rhubarb in the treatment of DN; 2) To identify the most effective ingredient of rhubarb for DN; 3) To explore the specific mechanism of rhubarb in treating DN.

**Methods:** Data sources: related studies were identified by searching Cochrane Library, Ovid-EMBASE, PubMed, SinoMed, WanFang, VIP, CNKI, and other Chinese magazines.

Assessment and analysis: SYRCLE’s risk of bias tool for animal studies was used to assess the quality of articles. The meta-analysis was performed in accordance with the Cochrane Handbook for Systematic Reviews of Interventions. Data analysis adopted RevMan 5.3 and STATA 12.0 software.

This study was published in the register with PROSPERO, number CRD42020204701.

**Results:** Aggregated data were collected from 27 eligible studies. The results illustrated an intense improvement in the following outcomes in rhubarb-treated animals with DN (*p* < 0.05): blood glucose, serum creatinine (Scr), blood urea nitrogen (BUN), albumin creatinine ratio (ACR), urine protein (UP), urinary albumin excretion (UAE), renal index (two kidneys weight/body weight, KW/BW), tubulointerstitial injury index (TII), transforming growth factor-beta1 (TGF-β1) mRNA and protein, alpha-smooth muscle actin (α-SMA) protein, and E-cadherin (E-cad) protein. Of these, DN animals with rhubarb exhibited a significantly higher level of E-cad protein. In addition, the level of the other outcomes mentioned above decreased significantly, while there was no significant association between the intervention and nephrin protein (*p* > 0.05).

**Conclusion:** This systematic review and meta-analysis demonstrated that rhubarb has a positive therapeutic effect on animals with DN, which may provide confidence and some theoretical reference for clinical application to a certain extent.

## Introduction

Diabetic nephropathy (DN) has been attracting much attention due to its increasing incidence (Perkovic et al., 2016). It is estimated that 463 million grown-ups suffer from diabetes worldwide, which is expected to increase to 700 million by 2045 (Saeedi et al., 2019). It is well known that DN is one of the most important microangiopathy in diabetes. In the United States and other developed countries, DN has become the first cause of end-stage renal disease. In the United States, the number of DN patients progressing to renal failure is projected to increase from 62,020 to 90,390 between 2015 and 2030 (Rowley et al., 2017). Besides, in China, the DN prevalence is also increasing year by year, and it accounts for 27% of the causes of chronic kidney disease (Wang et al., 2019a). As a severe complication of diabetes, its treatment has been a hot spot in many studies. Current studies showed that the pathogenesis of DN included genetic factors, and the following nongenetic factors: glucose and lipid (Chen et al., 2019) metabolism disorder, renal hemodynamic change (Hirukawa et al., 2018), cytokines (Xue, 2019), oxidative stress (Wang et al., 2019c), and inflammation (Nitta et al., 2018). At present, there are plenty of therapies aimed at pathogenesis, such as hypoglycemic agents, lipid-lowering drugs, angiotensin-converting enzyme inhibitors (ACEI), and protein kinase C-β (PKC-β) inhibitor (Fernandez-Fernandez et al., 2014). However, the curative effect and security of some drugs are not satisfactory. Due to the extensive pharmacological effects of traditional Chinese medicine, many scholars have extracted its effective components to study its curative effect and therapeutic mechanism. In recent years, traditional Chinese medicine has achieved a certain effect on the treatment of DN.

According to Chinese Pharmacopoeia (Commission, 2015), rhubarb is derived from the dried root and rhizome of *Rheum palmatum* L.*, Rheum offcinale* Baill*., and Rheum tanguticum* Maxim. ex Balf. Rhubarb was formerly used as a laxative, and recent researchers demonstrated that it has a beneficial effect on DN. Rhubarb is not only effective on DN animals, but also plays a certain therapeutic role in patients with DN. A clinical trial conducted by Li et al. (2011) indicated that proteinuria in DN patients treated with rhubarb combined with telmisartan was significantly lower than that in DN patients treated with telmisartan alone. Besides, treatment of rhubarb combined with telmisartan protected renal function by reducing blood viscosity and improving dyslipidemia. The constituents of rhubarb include free anthraquinones and compound anthraquinones (Ren et al., 2016). As for emodin (1,3,8-trihydroxy-6-methylanthraquinone) and rhein (1,8-dihydroxy-3-carboxyl anthraquinone), both of which are free anthraquinones, play an essential role in the treatment of diseases. It is known that rhein can alleviate the inflammation injury of the kidney; the inflammation of mouse kidney epithelial cell line induced with uric acid can be improved by rhein, which may be achieved by regulating lincRNA-Cox2 (Hu et al., 2020). Rhein can also improve chronic kidney disease of SD rats with 5/6 nephrectomy, this effect may be related to the regulation of the SIRT3/FOXO3α signaling pathway (Wu et al., 2020). In addition, rhein has the effect of attenuating blood glucose and reducing lipid peroxidation in DN mice (Li and Zhen, 2017). There is no significant effect of emodin on decreasing blood glucose, but it has a protective effect on kidney by reducing lipid, inflammation factors, and blood viscosity (Li et al., 2018).

Through a systematic review and meta-analysis, Hu et al. (2019) suggested that rhein has positive effects on DN animals, but it is not clear whether rhein is the most important component of rhubarb in the treatment of DN. Then which is the most effective ingredient of rhubarb in treating DN? Will the combined action of rhubarb components bring different curative effects? The objective of our systematic review and meta-analysis is to explore the efficacy and active constituents of rhubarb for DN and to find out the specific mechanisms of rhubarb in treating DN. Our analysis may provide scientific reference for the clinical application of rhubarb for DN.

## Methods

### Search Strategies

#### Electronic Databases Searching

We searched the following electronic bibliographic databases: Cochrane Library, Ovid-EMBASE, PubMed, SinoMed, WanFang, VIP, and CNKI. The publication dates chosen were from the establishment of the database to August 1st, 2020, and the languages were Chinese and English.

#### Manual Searching

We searched the following: Chinese Journal of Diabetes, Chinese Journal of Integrated Traditional and Western Nephrology, Chinese Journal of Nephrology, China Journal of Traditional Chinese Medicine and Pharmacy, Journal of Traditional Chinese Medicine, Chinese Journal of Integrated Traditional and Western Medicine, Journal of Clinical Nephrology, Chinese Journal of Nephrology, Dialysis and Transplantation, and Modern Journal of Integrated Traditional Chinese and Western Medicine. Publication date: From the establishment of the database to August 1st, 2020.

#### MeSH Terms With Free Words

According to the PRISMA 2020 statement (Page et al., 2021), we presented full search strategies for all databases. In English databases like Cochrane Library, Ovid-EMBASE, PubMed, and SinoMed, the related terms were used for the search: Participants (“Diabetic Nephropathies [MeSH],” “Nephropathies, Diabetic,” “Nephropathy, Diabetic,” “Diabetic Nephropathy,” “Diabetic Kidney Disease,” “Diabetic Kidney Diseases,” “Kidney Disease, Diabetic,” “Kidney Diseases, Diabetic,” “Diabetic Glomerulosclerosis,” “Kimmelstiel-Wilson Syndrome,” “Kimmelstiel Wilson Syndrome,” “Syndrome, Kimmelstiel-Wilson,” “Kimmelstiel-Wilson Disease,” “Kimmelstiel Wilson Disease,” “Nodular Glomerulosclerosis,” “Glomerulosclerosis, Nodular,” “Glomerulosclerosis, Diabetic,” “Intracapillary Glomerulosclerosis,” “DKD,” and “DN”). Intervention (“Rheum [MeSH],” “Rhubarb,” “Rheum officinale,” “Da Huang,” “Huang, Da,” “Chinese Rhubarb,” “Rhubarb, Chinese,” “Rheum rhaponticum,” “Rheum tanguticum,” “Emodin [MeSH],” and “Rhein”). Moreover, in Chinese databases like WanFang, VIP, and CNKI, the terms were adjusted for the search according to the Chinese corresponding to the above terms.

### Study Selection

The inclusion criteria were as follows: 1) participants: the diabetic nephropathy animal model (rats or mice, regardless of sex, age or weight, and the modeling method) was induced to meet the standard of diabetic nephropathy, blood glucose ≥16.65 mmol/L; 2) interventions: rhubarb (contained rhubarb, emodin, and rhein) was given by gavage, without limited administration period, frequency, and dose; 3) control: vehicle-treated animals, sham-treated animals (saline or distilled water), and animals undergoing no treatment; 4) primary outcomes: blood glucose, hemoglobin A1c (HbA1c), serum creatinine (Scr), blood urea nitrogen (BUN), albumin creatinine ratio (ACR), urine protein (UP), and urinary albumin excretion (UAE); secondary outcomes: kidney index (two kidneys weight/body weight, KW/BW), tubulointerstitial injury index (TII), transforming growth factor-beta 1 (TGF-β1) mRNA, TGF-β1 protein, alpha-smooth muscle actin (α-SMA) mRNA, α-SMA protein, E-cadherin (E-cad) mRNA, E-cad protein, podocin mRNA, podocin protein, nephrin mRNA, and nephrin protein; 5) study designs: randomized controlled trials and controlled studies with a separate control group; 6) language: Chinese and English.

The exclusion criteria were as follows: 1) participants: other animal models except for rats and mice (e.g., patients, *in vitro* cells, rabbits, dogs, and other animal models); 2) interventions: animals with rhubarb compound treatment, or animals in which rhubarb was administrated in other ways (such as intravenous injection or intraperitoneal injection); 3) control: animals with Western medicine, other traditional Chinese medicine, or integrated Chinese and Western medicine; 4) outcomes: other diabetic nephropathy outcomes; 5) study designs: semi-randomized or non-randomized controlled trials; 6) multiple publications (selected the one with the most complete data and integrated non-overlapping parts of duplicated studies) and incomplete original data; 7) crossover studies, review, case report, case analysis, meta-analysis, patent, and studies without a separate control group; 8) languages other than Chinese and English (Supplementary Table S1).

For example, although this article (Wang et al., 2019d) met the requirements of randomization and control criteria and had the outcome indicators we needed, mice with blood glucose greater than 16.1 mmol/L were included in the experiment, which did not meet our requirements for blood glucose greater than 16.65 mmol/L, that is why it was excluded. And another article (Wang et al., 2019b) was excluded because it was administered intravenously, which did not meet the inclusion criteria.

### Data Extraction

Two reviewers independently extracted data from each article. We attempted to extract numerical data from tables, figures, texts, or graphs in included studies (data in graphs were extracted by Adobe Photoshop CS5, according to this article *Advanced methods of data extraction for continuous outcomes in meta-analysis* (Liu et al., 2017a). When data were not reported or unclear, we attempted to get in touch with authors by e-mail (if authors did not respond, we had to delete those studies with incomplete and unclear data). In case an outcome was measured at multiple time points, data from the time point where efficacy is highest were included.

The following items were extracted: 1) author and year of publication; 2) the characteristics of the animal model: age or weight, gender, species, and model methods; 3) the number of animals in experimental and control groups; 4) intervention of interest: administration dosage and period; 5) control: vehicle in detail; 6) primary outcomes: blood glucose, mmol/L (was recalculated from mg/dl); HbA1c, %; Scr, μmol/L (was recalculated from mg/dl); BUN, mmol/L (was recalculated from mg/dl); ACR, mg/mmol; UP, mg/d; UAE, μg/24 h(was recalculated from mg/24 h); 7) secondary outcomes: kidney index, 10^−3^; TII; TGF-β1 mRNA and protein; α-SMA mRNA and protein; E-cad mRNA and protein; podocin mRNA and protein; and nephrin mRNA and protein.

### Quality Assessment

The method for risk of bias and quality assessment was as follows: two reviewers performed quality assessment independently with SYRCLE’s risk of bias tool for animal studies, any disagreements of them would be discussed with a third reviewer.

There were a total of ten items or five bias in the assessment tool, which included the following items: 1) random sequence generation (selection bias); 2) baseline characteristics (selection bias); 3) allocation concealment (selection bias); 4) random housing (performance bias); 5) blinding of participants and personnel (performance bias); 6) random outcome assessment (detection bias); 7) blinding of outcome assessment (detection bias); 8) incomplete outcome data (attrition bias); 9) selective reporting (reporting bias); and (10) other bias.

### Strategy for Data Analysis

According to PROSPERO, a random-effects model would be used to account for anticipated heterogeneity due to the exploratory nature of animal studies. All of our outcomes were continuous variables, which were presented as mean ± standard deviation (SD). Standardized mean difference (SMD) was applied when the unit of data was different, even if the unit had been converted. In the meanwhile, weighted mean difference (WMD) was utilized if the unit was the same and the mean difference was small. The confidence interval (CI) was set at 95%, and a *p*-value that did not exceed and not equal to 0.05 was considered to be significant. Whenever a study contained multiple experimental groups and just one control group, we would divide the total number of control animals in the meta-analysis into several parts according to the number of experimental groups, while the mean and SD of data remained unchanged.

Rhubarb is a traditional Chinese medicine, and both rhein and emodin are one of components of rhubarb. To evaluate the efficacy of the drug on improving DN animals, rhein, emodin, and rhubarb were regarded as the same intervention, and the same outcome of the three drugs were combined and analyzed. After exploring the curative effect of drugs on improving DN in animals, comparisons were conducted, respectively, of outcome indicators of rhein-treated, emodin-treated, and rhubarb-treated groups in order to identify the most effective components in rhubarb.

Subgroup analysis and sensitivity analysis was used to evaluate heterogeneity. Subgroup analysis was performed for more than eight studies according to the following variables: dosage (low, d ≤ 50 mg/kg; medium, 50 mg/kg < d ≤ 100 mg/kg; and high, d > 100 mg/kg); drug types (rhein, emodin, and rhubarb); duration (t < 8 w, 8 w ≤ t < 16 w, and t ≥ 16 w); and species (SD rats, Wistar rats, and mice). Sensitivity analysis was assessed by separately leaving each study out and rerunning the analysis with data. Funnel plots and the Egger’s regression test were utilized to estimate the publication bias when at least twenty studies were included.

The meta-analysis, subgroup analysis, sensitivity analysis, and publication bias were conducted by RevMan 5.3 software. Sensitivity analysis and the Egger’s test were conducted by STATA 12.0 software.

## Results

### Study Inclusion

**FIGURE 1:**
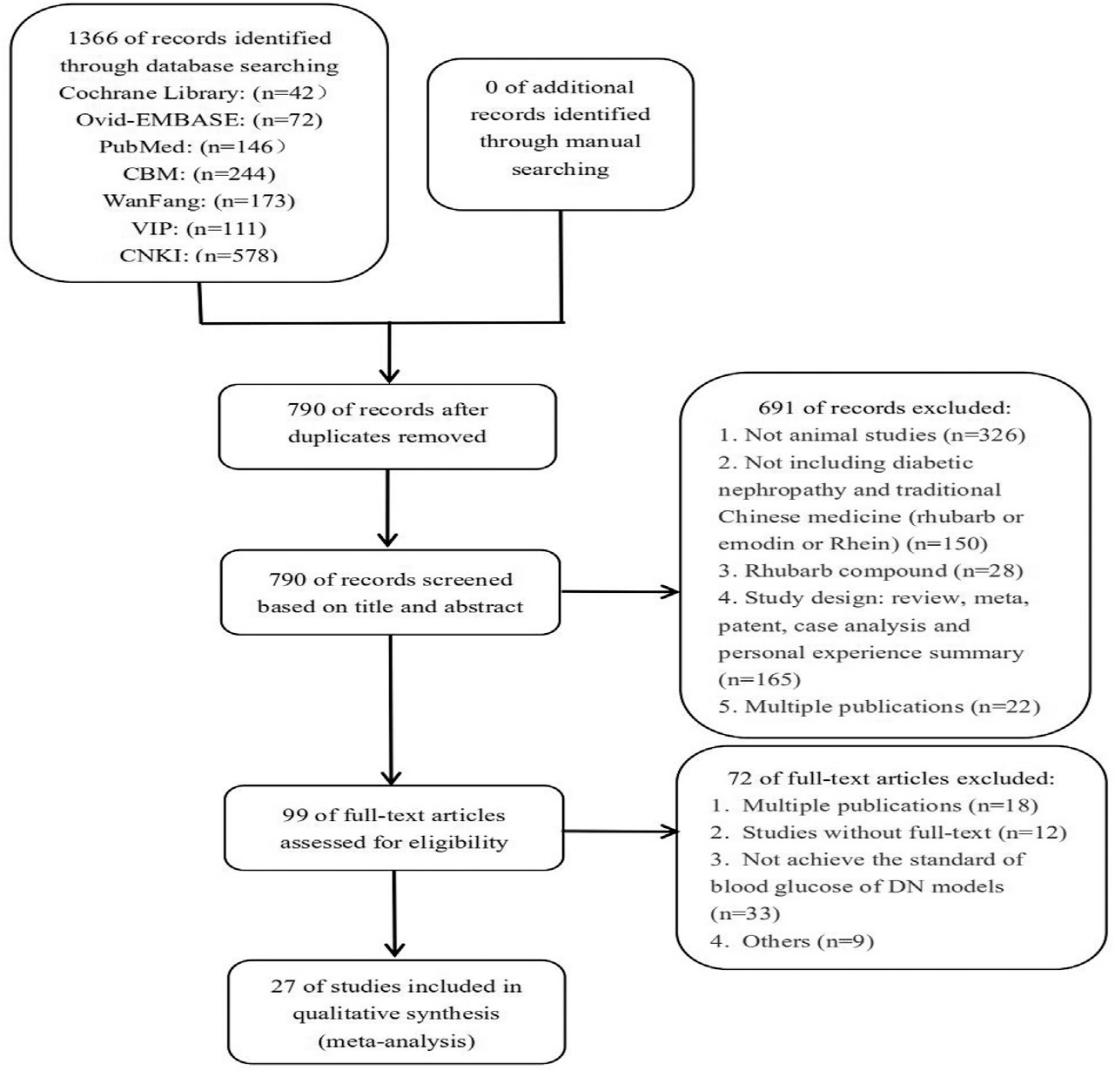
Study flow diagram.

A total of 790 records were identified through initial electronic databases searching. After duplicates were removed using EndNote software, the remaining 790 were screened according to their titles and abstracts. Then, 691 of records were excluded for the following reasons: 1) not animal studies; 2) not including diabetic nephropathy and traditional Chinese medicine (rhubarb or emodin or rhein); 3) rhubarb compound; 4) study design: review, meta-analysis, patent, case analysis, and personal experience summary; and 5) multiple publications. The remaining 99 records were reviewed. We excluded 72 records for the above reasons, with the addition of not meeting the inclusion criteria. Eventually, 27 studies were included in the analysis (Figure 1).

### Study Characteristics

There were 27 studies, which enrolled a total of 28 experiments and 731 animals in this systematic review and meta-analysis. Among them, the study by Tan (2002) contained two different experiments. The number of participants in the experimental group was 443 and that in the control group was 288. Included studies utilized three animal species, 11 studies of them (39%) used SD rats, 14 (50%) used Wistar rats, and three (11%) used mice. To establish a model with type 1 diabetic nephropathy, 19 of 28 studies (68%) used animals induced by streptozotocin (STZ). Two studies (7%) used animals induced by alloxan, and one study (4%) used both nephrectomy and STZ. To establish a model with type 2 diabetic nephropathy, four studies (14%) used STZ after high-fat-sugar diets and two studies (7%) used spontaneous obesity mice. The types of drugs included rhein, emodin, and rhubarb, animals in 14 studies (50%) took rhein. Seven studies (25%) took emodin and seven studies (25%) took rhubarb. There were three levels of dosage (low, medium, and high). Low dose (d ≤ 50 mg/kg) was used in nine studies (32%), and medium dose (50 mg/kg < d ≤ 100 mg/kg) was used in 18 studies (64%). Besides, high dose (d > 100 mg/kg) was used in 11 studies (39%). Nine of these studies used different levels of dosage simultaneously. We divided the administration time into short term, medium term, and long term. The animals in control groups were treated with 0.5% CMC-Na, saline, pure water, and distilled water. The characteristics of studies are summarized in Supplementary Table S2 (Ai et al., 2004; Liu and Ning, 2007; Jin et al., 2008; Wang, 2008; LI, 2008; Gao et al., 2010; LI, 2010; Hu et al., 2011; Wang et al., 2012; Chen et al., 2013; Hu et al., 2014; Chen, 2015; Chen et al., 2015b; Yang et al., 2020), Supplementary Table S3 (Zhu, 2006; Gao et al., 2014; Chen et al., 2015a; Yang et al., 2016; Jing et al., 2017; Shi, 2017; Tian et al., 2018), and Supplementary Table S4 (Yokozawa et al., 1997; Tan, 2003; Ni et al., 2004; Hamzeh et al., 2014; Li et al., 2017; Xue, 2019). The information about the medicine is displayed in Supplementary Table S5.

### Study Quality

**FIGURE 2:**
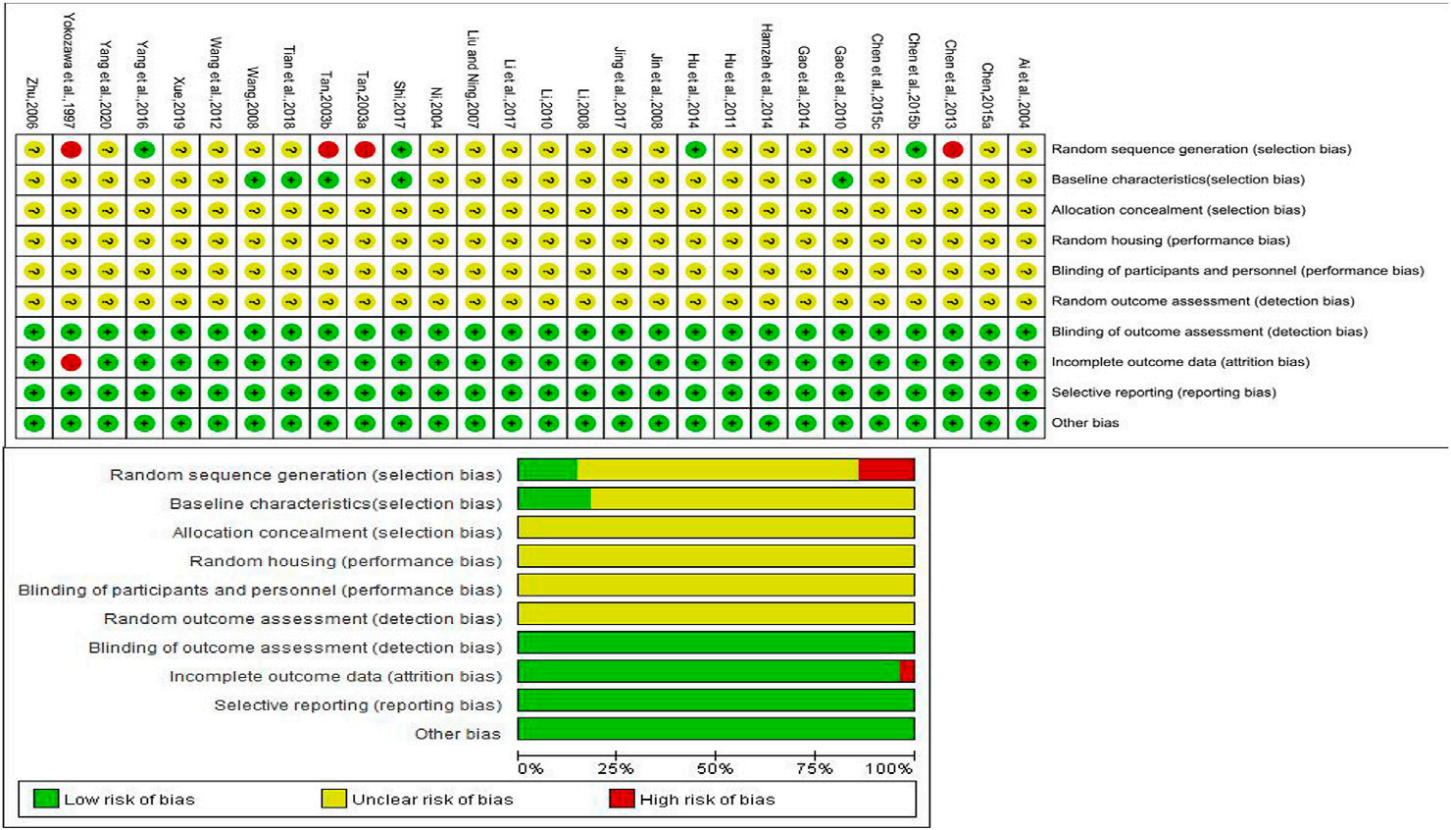
Risk of bias summary.

Twenty-four out of 28 studies mentioned random sequence generation, of which three studies mentioned the random number table method, and one mentioned a random sampling method. While the remaining four studies did not describe random allocation, which had a high risk of bias in random sequence generation (selection bias). Baseline characteristics of outcomes were illustrated in five studies. All studies did not describe the following items: allocation concealment (selection bias), random housing (performance bias), blinding of participants and personnel (performance bias), and random outcome assessment (detection bias). Blinding of outcome assessment (detection bias) was assessed as low risk of bias. Almost all studies reported complete outcome data but one (Yokozawa et al., 1997), which did not clarify the reason why the animals were missing. All anticipated results were reported and other biases did not exist. The results of SYRCLE’s risk of bias tool are provided in Figure 2.

### Effectiveness

#### Blood Glucose

**FIGURE 3:**
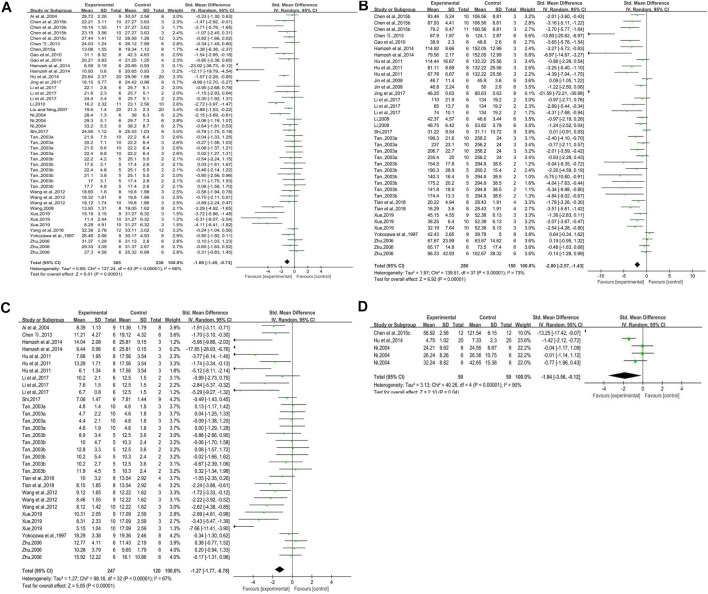
**(A)** Forest plot of blood glucose, **(B)** forest plot of Scr, **(C)** forest plot of BUN, and **(D)** forest plot of ACR.

Twenty-three studies (some studies included more than one intervention group) reported on blood glucose. There was a significant difference in blood glucose between the experimental groups and the control groups. Blood glucose decreased in DN animals with rhubarb (contained rhubarb, emodin, and rhein) compared with that in control groups. However, there were clear heterogeneity, we needed to conduct subgroup analysis to find out the origination of heterogeneity (*n* = 603, SMD = −1.09, 95% CI [−1.45, −0.73], *p* < 0.00001; heterogeneity: Chi^2^ = 127.24, *p* < 0.00001, *I* ^*2*^ = 66% Figure 3A).

#### Hemoglobin A1c

Only one study (Yang et al., 2016) reported on HbA1c; hence, a meta-analysis was impossible. This study contained 24 participants, the mean ± SD of blood glucose in the experimental group was 20.10 ± 3.3. While that in the control group was 18 ± 3.2. The intervention group showed the poorly controlled HbA1c levels.

#### Serum Creatinine

Sixteen studies reported on serum creatinine. The forest plot showed that rhubarb significantly reduced the creatinine level of the experimental groups. But as there was obvious heterogeneity, we were supposed to do subgroup analysis to find out the reason (*n* = 430, SMD = −2.00, 95% CI [−2.57, −1.43], *p* < 0.00001; heterogeneity: Chi^2^ = 139.51, *p* < 0.00001, *I* ^*2*^ = 73% Figure 3B).

#### Blood Urea Nitrogen

Thirteen studies reported the impact of rhubarb on blood urea nitrogen. The BUN in the experimental groups was significantly lower than that in the control groups. However, heterogeneity existed clearly, and we had to conduct subgroup analysis to find out the reason (*n* = 367, SMD = −1.27, 95% CI [−1.77, −0.78], *p* < 0.00001; heterogeneity: Chi^2^ = 98.16, *p* < 0.00001, *I* ^*2*^ = 67% Figure 3C).

#### Albumin Creatinine Ratio

Three studies reported on this outcome. This forest plot indicated that there was significant difference in ACR between the experimental groups and the control groups. The level of ACR decreased when animals were treated with rhubarb (*n* = 100, SMD = −1.84, 95% CI [−3.56, −0.12], *p* = 0.04; heterogeneity: Chi^2^ = 40.26, *p* < 0.00001, *I* ^*2*^ = 90% Figure 3D).

#### Urine Protein

**FIGURE 4:**
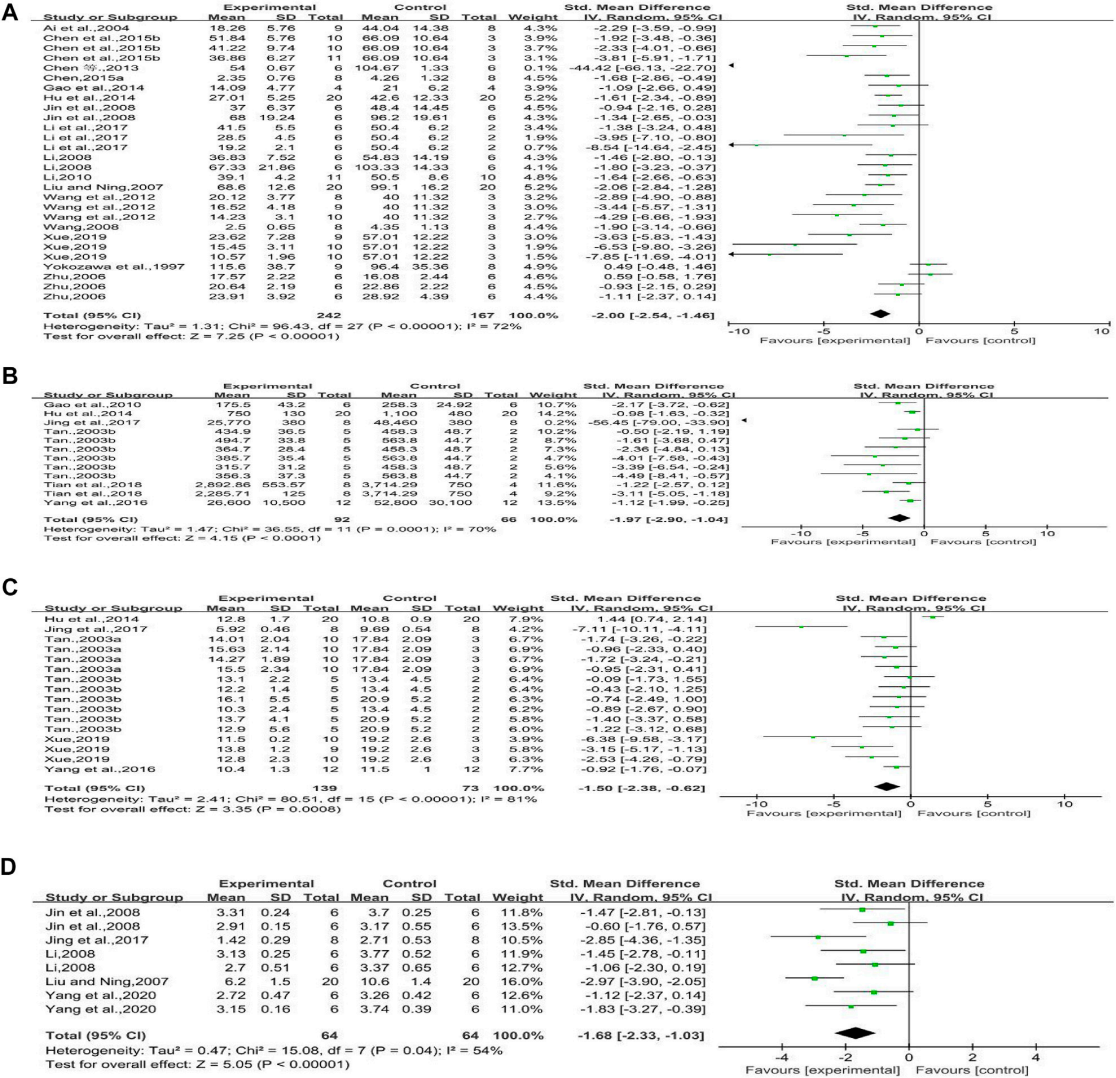
**(A)** Forest plot of UP, **(B)** forest plot of UAE, **(C)** forest plot of KW/BW, and **(D)** forest plot of TII.

Sixteen studies reported on urine protein. It could be learned from this forest plot that rhubarb could significantly improve the urine protein in the experimental groups compared with that in the control groups. However, there was clear heterogeneity, and we needed to conduct subgroup analysis to find out the reason (*n* = 409, SMD = −2.00, 95% CI [−2.54, −1.46], *p* < 0.00001; heterogeneity: Chi^2^ = 96.43, *p* < 0.00001, *I* ^*2*^ = 72% Figure 4A).

#### Urinary Albumin Excretion

Six studies reported on urinary albumin excretion. The forest plot showed that rhubarb noticeably reduced the level of UAE (*n* = 158, SMD = −1.97, 95% CI [−2.90, −1.04], *p* < 0.0001; heterogeneity: Chi^2^ = 36.55, *p* < 0.0001, *I* ^*2*^ = 70% Figure 4B).

#### Kidney Weight/Body Weight

Six studies reported the impact of rhubarb on kidney weight/body weight. As shown in Figure 4C, the intervention of rhubarb treatment could greatly reduce the kidney index (KW/BW) (*n* = 212, SMD = −1.50, 95% CI [−2.38, −0.62], *p* = 0.0008; heterogeneity: Chi^2^ = 80.51, *p* < 0.00001, *I* ^*2*^ = 81% Figure 4C).

#### Tubulointerstitial Injury Index

Five studies reported on the tubulointerstitial injury index. Rhubarb showed intensive effects on decreasing the level of tubulointerstitial injury index (*n* = 128, SMD = −1.68, 95% CI [−2.33, −1.03], *p* < 0.00001; heterogeneity: Chi^2^ = 15.08, *p* = 0.04, *I* ^*2*^ = 54% Figure 4D).

#### Transforming Growth Factor-Beta1 mRNA

**FIGURE 5:**
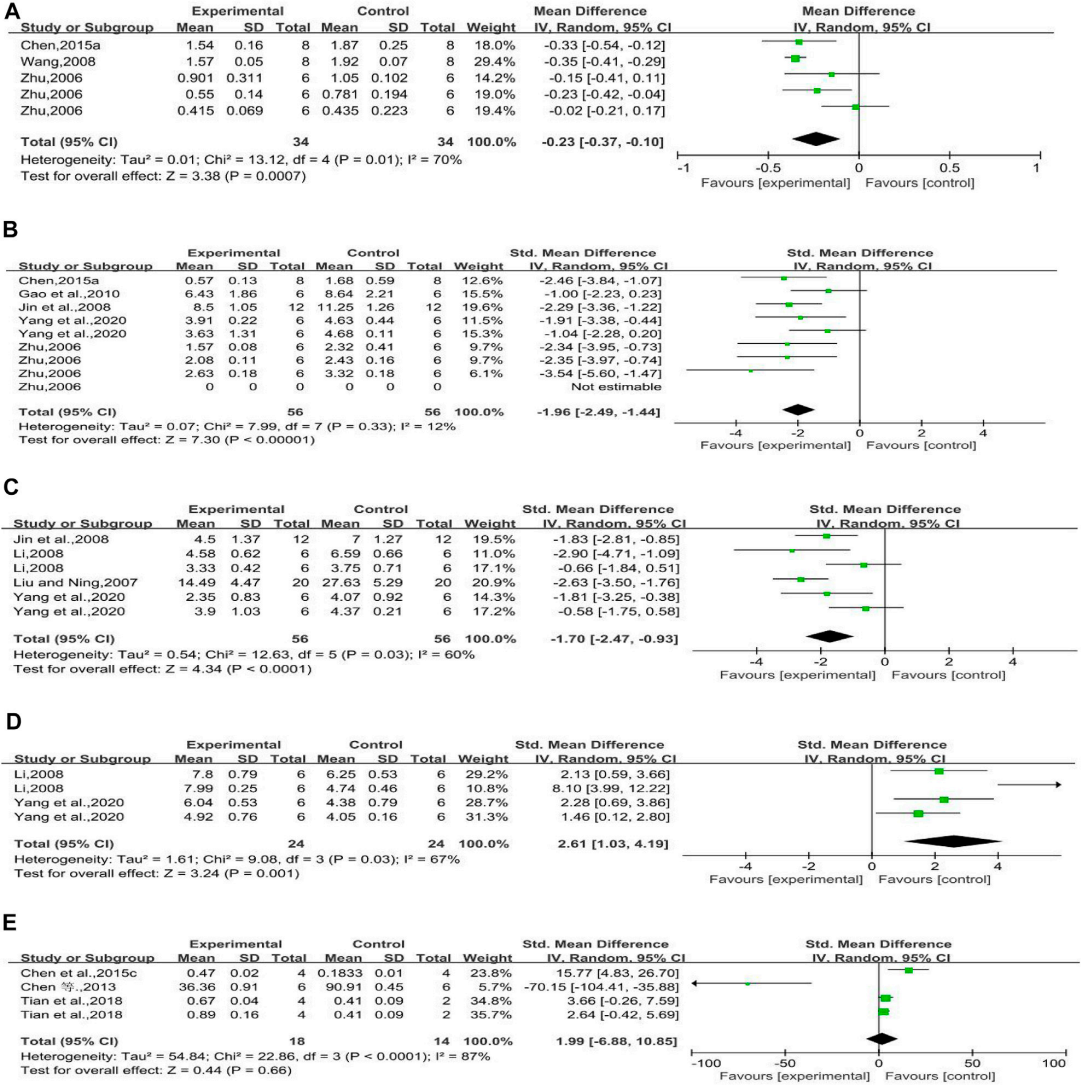
**(A)** Forest plot of TGF-β1 mRNA, **(B)** forest plot of TGF-β1 protein, **(C)** forest plot of α-SMA protein, **(D)** forest plot of E-cad protein, and **(E)** forest plot of nephrin protein.

Three studies reported the effect of rhubarb on this outcome. It seemed that rhubarb applied to experimental groups could significantly reduce TGF-β1 mRNA (*n* = 68, MD = −0.23, 95% CI [−0.37, −0.10], *p* = 0.0007; heterogeneity: Chi^2^ = 13.12, *p* = 0.01, *I* ^*2*^ = 70% Figure 5A).

#### Transforming Growth Factor-Beta1 Protein

Five studies reported the effect of rhubarb on TGF-β1 protein. It seemed that rhubarb applied to experimental groups could significantly decrease TGF-β1 protein, but without significant heterogeneity (*n* = 112, SMD = −1.96, 95% CI [−2.49,−1.44], *p* < 0.00001; heterogeneity: Chi^2^ = 7.99, *p* = 0.33, *I* ^*2*^ = 12% Figure 5B).

#### Alpha-Smooth Muscle Actin Protein

Four studies reported on this outcome. The forest plot showed that rhubarb noticeably reduced α-SMA protein compared with that in the control group (*n* = 112, SMD = −1.70, 95% CI [−2.47, −0.93], *p* < 0.0001; heterogeneity: Chi^2^ = 12.63, *p* = 0.03, *I* ^*2*^ = 60% Figure 5C).

#### E-cadherin Protein

Two studies reported on this outcome. When compared to the E-cad protein of control groups, that of experimental groups with rhubarb noticeably increased (*n* = 48, SMD = 2.61, 95% CI [1.03, 4.19], *p* = 0.001; heterogeneity: Chi^2^ = 9.08, *p* = 0.03, *I* ^*2*^ = 67% Figure 5D).

#### Nephrin Protein

Three studies reported on this outcome. The forest plot showed that rhubarb did not significantly increase nephrin protein compared with that in the control groups. In a normal condition, nephrin expression is increased with the improvement of diabetic nephropathy. One of these included studies (Chen et al., 2013) indicated that rhein has an inhibitory effect on the expression of podocyte nephrin. The reason for this study’s conclusion still need to be further studied (*n* = 32, SMD = 1.99, 95% CI [−6.88, 10.85], *p* = 0.66; heterogeneity: Chi^2^ = 22.86, *p* < 0.0001, *I* ^*2*^ = 87% Figure 5E).

#### Other Outcome

There were no data on α-SMA mRNA, E-cad mRNA, Podocin mRNA and protein, and nephrin mRNA in all studies.

### Subgroup Analysis

1) Blood glucose: Subgroup analysis of blood glucose showed that the curative effect of rhubarb in Wistar rats was better than that in SD rats and mice (*p* = 0.02). However, there was no significant difference between the subgroup of dosage (*p* = 0.05), drug types (*p* = 0.21), and duration (*p* = 0.14) (Supplementary Table S6).
2) Scr: There was no significant difference in subgroup analysis according to dosage (*p* = 0.43), drug types (*p* = 0.30), duration (*p* = 0.23), and species (*p* = 0.82) (Supplementary Table S7).
3) BUN: As shown in Supplementary Table S7, the efficacy of rhein on improving this outcome was better compared with that of emodin and rhubarb (*p* = 0.0004). Besides, medicine significantly decreased BUN in Wistar rats than that in SD rats and mice (*p* = 0.04). But there was no significant difference in subgroup analysis based on dosage (*p* = 0.12) and duration (*p* = 0.25) (Supplementary Table S8).
4) UP: It could be learned from Supplementary Table S8 that eight to sixteen weeks was the best treatment duration (*p* = 0.01), and the efficacy of rhubarb on urine protein of diabetic nephropathy animals was more effective than that of rhein and emodin (*p* = 0.003), while other subgroup analyses, which were based on dosage (*p* = 0.06) and species (*p* = 0.41), did not perform a significant difference (Supplementary Table S9).

### Sensitivity Analysis

Four outcomes were included in the sensitivity analysis.

#### Blood Glucose

We assessed a sensitivity analysis of blood glucose by separately leaving each one study out and pooling an estimate of blood glucose. As a result, no change was observed.

#### Serum Creatinine

We assessed a sensitivity analysis of serum creatinine by leaving each one study out and observing no change in these results.

#### Blood Urea Nitrogen

A sensitivity analysis of blood urea nitrogen was conducted by separately excluding each one study and making a summary and evaluation of this outcome. There was no change in these results.

#### Urine Protein

A sensitivity analysis indicated that the results were stable and reliable.

### Publication Bias

**FIGURE 6:**
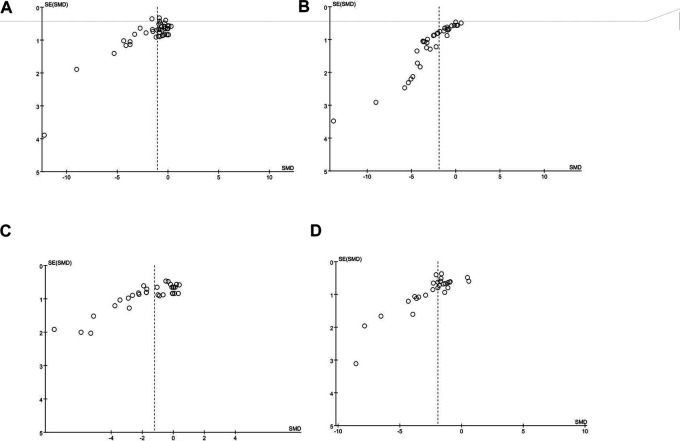
**(A)** Funnel plot of blood glucose, **(B)** funnel plot of Scr, **(C)** funnel plot of BUN, and **(D)** funnel plot of UP.

The publication bias of these studies was assessed by the funnel plot. We learned from this funnel plot that publication bias may have existed (Figure 6). The results of the following four outcomes, which were judged by the funnel plots, were supported by the Egger’s test: blood glucose (*p* < 0.05), Scr (*p* < 0.05), BUN (*p* < 0.05), and UP (*p* < 0.05) (Supplementary Table S10).

### Further Analysis

#### Further Effectiveness Analysis

Blood glucose: It could be considered that the blood glucose level in the experimental group treated with rhein was lower than that in the control group (*n* = 250, SMD = −1.48, 95% CI [−2.02, −0.94], *p* < 0.00001; heterogeneity: Chi^2^ = 35.98, *p* = 0.0006, *I* ^*2*^ = 64% Supplementary Figure S1A). However, the forest plot showed that the level of blood glucose did not decrease in emodin-treated group compared with that in the control group (*n* = 126, SMD = −0.71, 95% CI [−1.48, 0.05], *p* =0.07; heterogeneity: Chi^2^ = 24.61, *p* = 0.0009, *I* ^*2*^ = 72% Supplementary Figure S1B). Besides, rhubarb could reduce blood glucose level compared with that in the control group (*n* = 227, SMD = −0.96, 95% CI [−1.53, −0.38], *p* = 0.001; heterogeneity: Chi^2^ = 55.41, *p* < 0.0001, *I* ^*2*^ = 62% Supplementary Figure S1C).

Scr: Scr decreased in DN animals with rhein (*n* = 145, SMD = −2.20, 95% CI [−3.15, −1.26], *p* < 0.00001; heterogeneity: Chi^2^ = 40.44, *p* < 0.0001, *I* ^*2*^ = 73% Supplementary Figure S2A). However, Scr did not decrease in DN animals with emodin (*n* = 94, SMD =−1.05, 95% CI [−2.41, 0.31], *p* = 0.13; heterogeneity: Chi^2^ = 37.38, *p* < 0.00001, *I* ^*2*^ = 84% Supplementary Figure S2B). As for animals treated with rhubarb, Scr decreased in DN animals with rhubarb compared with that in control groups (*n* = 191, SMD = −2.25, 95% CI [−3.05, −1.44], *p* < 0.00001; heterogeneity: Chi^2^ = 49.17, *p* = 0.0001, *I* ^*2*^ = 63% Supplementary Figure S2C).

BUN: As shown in Supplementary Figure S3, there was no obvious heterogeneity in the outcome of animals with rhein and emodin, while there was moderate heterogeneity in the outcome of animals with rhubarb. The intervention of rhein treatment could reduce the level of BUN (*n* = 98, SMD = −2.18, 95% CI [−2.76, −1.59], *p* < 0.00001; heterogeneity: Chi^2^ = 6.95, *p* = 0.43, *I* ^*2*^ = 0% Supplementary Figure S3A). However, emodin treatment could not lower the level of BUN (*n* = 78, SMD = −0.44, 95% CI [−1.09, 0.20], *p* = 0.18; heterogeneity: Chi^2^ = 8.87, *p* = 0.11, *I* ^*2*^ = 44% Supplementary Figure S3B). And the intervention of rhubarb could reduce the level of BUN (*n* = 191, SMD = −1.18, 95% CI [−1.92, −0.43], *p* = 0.002; heterogeneity: Chi^2^ = 58.07, *p* < 0.00001, *I* ^*2*^ = 69% Supplementary Figure S3C).

UP: There was obviously association of rhein with UP (*n* = 286, SMD = −2.01, 95% CI [−2.47, −1.55], *p* < 0.00001; heterogeneity: Chi^2^ = 30.06, *p* = 0.02, *I* ^*2*^ = 47% Supplementary Figure S4A). However, emodin did not significantly reduce UP compared with that in control groups (*n* = 44, SMD = −0.58, 95% CI [−1.44, 0.27], *p* = 0.18; heterogeneity: Chi^2^ = 5.25, *p* = 0.15, *I* ^*2*^ = 43% Supplementary Figure S4B). As for animals treated with rhubarb, UP decreased in DN animals with rhubarb compared with that in control groups (*n* = 79, SMD = −3.95, 95% CI [−6.48, −1.42], *p* = 0.002; heterogeneity: Chi^2^ = 46.51, *p* < 0.00001, *I* ^*2*^ = 87% Supplementary Figure S4C).

#### Further Subgroup Analysis

1) Blood glucose in rhein-treated group: There was no significant difference between the subgroup of dosage (*p* = 0.75), duration (*p* = 0.14), and species (*p* = 0.83).
2) Scr in the rhein-treated group: There was no significant difference between the subgroup of duration (*p* = 0.22) and species (*p* = 0.14). However, the efficacy of rhein was better when the dosage was more than 100 mg/kg (*p* = 0.02) (Supplementary Table S11).
3) Blood glucose in emodin-treated group: There was no significant difference between the subgroup of dosage (*p* = 0.51), duration (*p* = 0.39), and species (*p* = 0.33).
4) Scr in emodin-treated group: There was no significant difference between the subgroup of dosage (*p* = 0.44) and duration (*p* = 0.73). As for the species of animals, the difference of animal species might be one of the sources of heterogeneity (*p* = 0.02) (Supplementary Table S12).
5) Blood glucose in rhubarb-treated group: Rhubarb had different therapeutic effects on different kinds of animals. After rhubarb treatment, the blood glucose of Wistar rats could be improved better than that of SD rats and mice (*p* = 0.03). But there was no significant association correlation of rhubarb with blood glucose for the subgroup of dosage (*p* = 0.22) and duration (*p* = 0.16).
6) Scr in the rhubarb-treated group: There was no significant difference between the subgroup of dosage (*p* = 0.11), duration (*p* = 0.47), and species (*p* = 0.14).
7) BUN in the rhubarb-treated group: Rhubarb had different therapeutic effects on different kinds of animals. After rhubarb treatment, the BUN of Wistar rats could be improved better (*p* = 0.01). But there was no significant association correlation of rhubarb with BUN for the subgroup of dosage (*p* = 0.12) and duration (*p* = 0.10) (Supplementary Table S13).

#### Sensitivity Analysis

We analyzed separately the four outcomes (blood glucose, Scr, BUN, and UP) of three different interventions in the sensitivity analysis. The sensitivity analysis indicated that the results were stable and reliable.

## Discussion

It could be inferred from this systematic review and meta-analysis that rhubarb played a beneficial role in the treatment of animals with DN. After regarding the three drugs as the same intervention, and aggregating and analyzing data of the same outcome of the three drugs, we found that rhubarb could improve blood glucose, Scr, BUN, UP, and UAE, which was related to reducing TGF-β1, α-SMA, and improving E-cad. We demonstrated substantial statistical heterogeneity for ACR and KW/BW and moderate heterogeneity for blood glucose, Scr, BUN, UP, and UAE. Part of this heterogeneity might originate from the differences in pooled studies including pharmacologic differences and variations in animal species and basic design.

To find out and eliminate the source of heterogeneity, and to compare which drug is more effective, we grouped the outcome indicators according to rhein, emodin, and rhubarb and further processed the data. In view of the results of further effectiveness, it was considered that rhein, as the most effective components of rhubarb, could significantly improve DN. However, emodin treatment could not improve DN. There was no statistically significant difference between emodin-treated group and control group in reducing blood glucose, Scr, BUN, and UP. Thus, the association between emodin and outcome indicators might be overestimated when the data of rhein, emodin, and rhubarb were used together to evaluate the efficacy. The previous meta-analysis conducted by Hu et al. indicated that the levels of blood glucose, Scr, and UP in DN animals significantly decreased after administration of rhein. However, from the subgroup analysis of this meta-analysis, rhein significantly improved the BUN of DN animals compared with rhubarb and emodin, while rhubarb had a stronger effect on reducing urine protein of DN animals than rhein and emodin.

Glucose and lipid metabolism disorders are one of the most important pathogenesis of DN. The levels of blood glucose and HbA1c reflect the condition of glucose metabolism to some extent. The renal index indicates the degree of renal hypertrophy and reflects the level of lipid metabolism. Rhubarb has a hypoglycemic effect; however, Yang (Yang et al., 2016) found that the HbA1c increased after treatment. It might be due to the fact that HbA1c reflects the long-term control of blood glucose within three months, which was not well controlled in rats of this study.

These outcomes, including Scr, BUN, ACR, UP, and UAE, can reflect renal function. Rhubarb has a protective effect on renal function. There was no significant difference in subgroup analysis of Scr according to dosage (*p* > 0.05), drug types (*p* > 0.05), duration (*p* > 0.05), and species (*p* > 0.05). However, in subgroup analysis, the Scr of rhein treatment group decreased significantly when the rhein dose exceeded 100 mg/kg (*p* = 0.02). The difference between drug types and species made a difference to BUN. Compared with the BUN of animals treated with other drugs, the BUN of animals treated with rhein was significantly improved. And the BUN of Wistar rats was also clearly improved compared with that of other species. For UP, different types of drugs and administration duration had different therapeutic effects. The UP in the rhubarb-treated groups was a lower level than that in rhein-treated and emodin-treated groups. As for ACR and UAE, rhubarb noticeably reduced their levels. We speculate that rhein may be more suitable for DN animals with increased BUN, while rhubarb may be more effectively for DN animals with elevated UP. It is unclear whether the findings observed in DN animals can also be observed in patients. Therefore, the clinical efficacy, appropriate dosage, treatment course of rhubarb, and its ingredients need to be further explored.

In the process of renal fibrosis, epithelial–mesenchymal transdifferentiation (EMT) in renal tubule cells and injury in podocytes are of great significance (Kalluri et al., 2015). The degree of EMT could be perceived from the change of the following indicators: TII, TGF-β1, α-SMA, and E-cadherin, while the damage of podocytes could be judged by the levels of podocin and nephrin. It seemed that rhubarb applied to experimental groups could significantly reduce renal fibrosis by decreasing the degree of TII, TGF-β1 mRNA and protein, and α-SMA protein, and increasing the degree of E-cad protein. In this study, whether rhubarb has a protective effect on podocytes is still questionable due to the lack of data for evaluating the damage of podocytes.

We are not supposed to neglect the safety of rhubarb while evaluating its efficacy. Through observing rats with rhubarb anthraquinones for 90 days, Cheng (Cheng et al., 2020) concluded that rhubarb-treated rats could suffer from colonic toxicities, including melanosis coli. Liu et al., (2017b) found from a retrospective study that 206 out of 219 patients with melanosis coli had abused laxatives. Large doses or long-term use of rhubarb may lead to melanosis coli. Therefore, we should pay attention to the safe dose of rhubarb and timely reduction or stopping the drug according to the conditions of patients. We also need to closely monitor and protect organs that may be damaged by drugs.

In these studies, there were some limitations. First, the quality of the studies was not high enough because of non-random allocation and others. Second, as can be seen from the funnel plots, potential bias still existed in this analysis. Third, the units of included data were different, which means that the data might be measured in different ways. Finally, the lack of data on some outcomes such as podocin leads to the imperfection of this research.

## Conclusion

In summary, this systematic review and meta-analysis demonstrated that rhubarb has a positive therapeutic effect on animals with diabetic nephropathy. Rhubarb could reduce the level of blood glucose and renal index, thereby improving glucose and lipid metabolism disorder. It could also decrease the level of TGF-β1 and α-SMA and increase that of E-cad, regulating the proliferation and epithelial–mesenchymal transdifferentiation in renal tubule cells. However, potential bias still existed. To get more accurate and stable results of the analysis, more large-scale, high-quality, and rigorous-design studies are required.

## Data Availability

The original contributions presented in the study are included in the article/Supplementary Material, and further inquiries can be directed to the corresponding author.
