# Supplementary material for: The Effects of Rhubarb for the Treatment of Diabetic Nephropathy in Animals: A Systematic Review and Meta-analysis
Source: Front Pharmacol. 2021 Jun 11;12:602816. doi: 10.3389/fphar.2021.602816 (PMC8226322; doi:10.3389/fphar.2021.602816)
Supplement: Supplementary file 1 [file Image4.pdf]

Study or Subgroup	Experimental Mean	Experimental SD	Experimental Total	Control Mean	Control SD	Control Total	Weight	Std. Mean Difference IV, Random, 95% CI
Ai et al.,2004	18.26	5.76	9	44.04	14.38	8	6.7%	-2.29 [-3.59, -0.99]
Chen et al.,2013	54	0.67	6	104.67	1.33	6	0.0%	-44.42 [-66.13, -22.70]
Chen et al.,2015b	51.84	5.76	10	66.09	10.64	3	5.4%	-1.92 [-3.48, -0.36]
Chen et al.,2015b	41.22	9.74	10	66.09	10.64	3	4.9%	-2.33 [-4.01, -0.66]
Chen et al.,2015b	36.86	6.27	11	66.09	10.64	3	3.6%	-3.81 [-5.91, -1.71]
Chen,2015a	2.35	0.76	8	4.26	1.32	8	7.3%	-1.68 [-2.86, -0.49]
Hu et al.,2014	27.01	5.25	20	42.6	12.33	20	10.5%	-1.61 [-2.34, -0.89]
Jin et al.,2008	37	6.37	6	48.4	14.45	6	7.1%	-0.94 [-2.16, 0.28]
Jin et al.,2008	68	19.24	6	96.2	19.61	6	6.6%	-1.34 [-2.65, -0.03]
Li,2008	36.83	7.52	6	54.83	14.19	6	6.4%	-1.46 [-2.80, -0.13]
Li,2008	67.33	21.86	6	103.33	14.33	6	6.0%	-1.80 [-3.23, -0.37]
Li,2010	39.1	4.2	11	50.5	8.6	10	8.3%	-1.64 [-2.66, -0.63]
Liu and Ning,2007	68.6	12.6	20	99.1	16.2	20	10.0%	-2.06 [-2.84, -1.28]
Wang et al.,2012	20.12	3.77	8	40	11.32	3	3.8%	-2.89 [-4.90, -0.88]
Wang et al.,2012	16.52	4.18	9	40	11.32	3	3.5%	-3.44 [-5.57, -1.31]
Wang et al.,2012	14.23	3.1	10	40	11.32	3	3.0%	-4.29 [-6.66, -1.93]
Wang,2008	2.5	0.65	8	4.35	1.13	8	7.0%	-1.90 [-3.14, -0.66]
Yang et al.,2020	0	0	0	0	0	0		Not estimable
**Total (95% CI)**			**164**			**122**	**100.0%**	**-2.01 [-2.47, -1.55]**

Heterogeneity: Tau² = 0.39; Chi² = 30.06, df = 16 (P = 0.02); I² = 47%
Test for overall effect: Z = 8.57 (P < 0.00001)

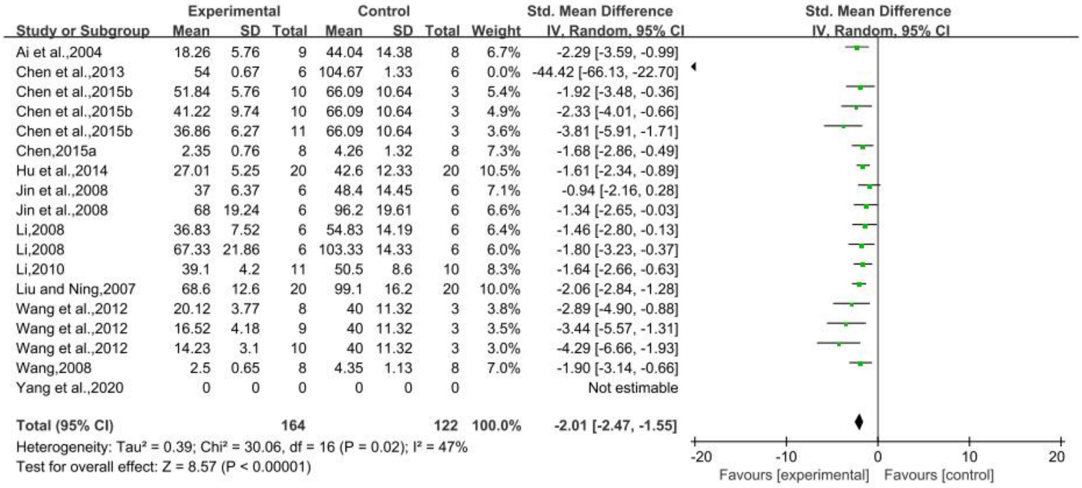

(A)

Study or Subgroup	Experimental Mean	Experimental SD	Experimental Total	Control Mean	Control SD	Control Total	Weight	Std. Mean Difference IV, Random, 95% CI
Gao et al.,2014	14.09	4.77	4	21	6.2	4	19.6%	-1.09 [-2.66, 0.49]
Zhu,2006	17.57	2.22	6	16.08	2.44	6	27.9%	0.59 [-0.58, 1.76]
Zhu,2006	20.64	2.19	6	22.86	2.22	6	26.7%	-0.93 [-2.15, 0.29]
Zhu,2006	23.91	3.92	6	28.92	4.39	6	25.8%	-1.11 [-2.37, 0.14]
**Total (95% CI)**			**22**			**22**	**100.0%**	**-0.58 [-1.44, 0.27]**

Heterogeneity: Tau² = 0.32; Chi² = 5.25, df = 3 (P = 0.15); I² = 43%
Test for overall effect: Z = 1.34 (P = 0.18)

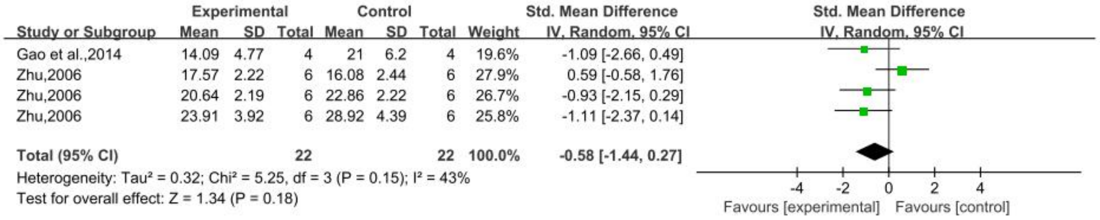

(B)

Study or Subgroup	Experimental Mean	Experimental SD	Experimental Total	Control Mean	Control SD	Control Total	Weight	Std. Mean Difference IV, Random, 95% CI
Li et al.,2017	41.5	5.5	6	50.4	6.2	2	16.6%	-1.38 [-3.24, 0.48]
Li et al.,2017	28.5	4.5	6	50.4	6.2	2	14.2%	-3.95 [-7.10, -0.80]
Li et al.,2017	19.2	2.1	6	50.4	6.2	2	8.8%	-8.54 [-14.64, -2.45]
Xue,2019	23.62	7.28	9	57.01	12.22	3	16.0%	-3.63 [-5.83, -1.43]
Xue,2019	15.45	3.11	10	57.01	12.22	3	13.9%	-6.53 [-9.80, -3.26]
Xue,2019	10.57	1.96	10	57.01	12.22	3	12.8%	-7.85 [-11.69, -4.01]
Yokozawa et al.,1997	115.6	38.7	9	96.4	35.36	8	17.7%	0.49 [-0.48, 1.46]
**Total (95% CI)**			**56**			**23**	**100.0%**	**-3.95 [-6.48, -1.42]**

Heterogeneity: Tau² = 9.13; Chi² = 46.51, df = 6 (P < 0.00001); I² = 87%
Test for overall effect: Z = 3.07 (P = 0.002)

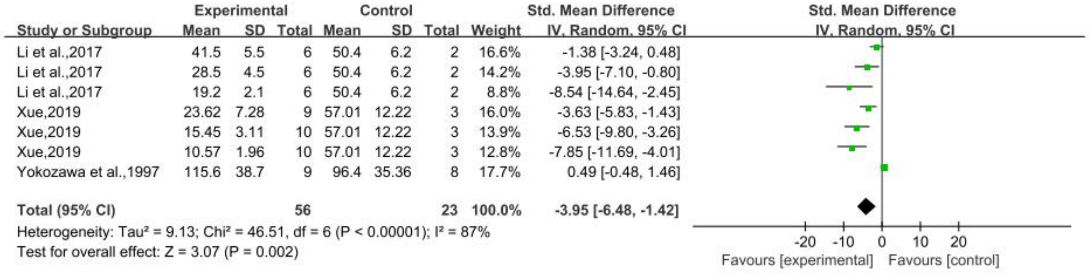

(C)

Figure S4 (A)Forest plot of UP with rhein; (B)Forest plot of UP with emodin; (C)Forest plot of UP with rhubarb
