# Supplementary material for: The Effects of Rhubarb for the Treatment of Diabetic Nephropathy in Animals: A Systematic Review and Meta-analysis
Source: Front Pharmacol. 2021 Jun 11;12:602816. doi: 10.3389/fphar.2021.602816 (PMC8226322; doi:10.3389/fphar.2021.602816)
Supplement: Supplementary file 2 [file Image2.pdf]

Study or Subgroup	Experimental Mean	Experimental SD	Experimental Total	Control Mean	Control SD	Control Total	Weight	Std. Mean Difference IV, Random, 95% CI
Chen et al.,2013	87.9	1.87	6	124.1	2.87	6	1.7%	-13.80 [-20.62, -6.97]
Chen et al.,2015b	93.46	5.24	10	106.56	8.81	3	9.3%	-2.01 [-3.60, -0.43]
Chen et al.,2015b	87.93	4.41	10	106.56	8.81	3	8.2%	-3.16 [-5.11, -1.22]
Chen et al.,2015b	79.2	6.47	11	106.56	8.81	3	7.9%	-3.70 [-5.77, -1.64]
Gao et al.,2010	38.9	2.3	6	48.6	2.6	6	7.7%	-3.65 [-5.76, -1.54]
Hu et al.,2011	114.44	16.67	8	132.22	25.56	3	9.9%	-0.86 [-2.26, 0.54]
Hu et al.,2011	81.11	8.89	8	132.22	25.56	3	7.6%	-3.25 [-5.40, -1.10]
Hu et al.,2011	67.78	6.67	8	132.22	25.56	3	6.3%	-4.39 [-7.04, -1.75]
Jin et al.,2008	46.7	11.4	6	45.9	3.6	6	10.7%	0.09 [-1.05, 1.22]
Jin et al.,2008	46.8	2.24	6	50	2.6	6	10.2%	-1.22 [-2.50, 0.06]
Li,2008	42.37	4.57	6	46.6	3.44	6	10.4%	-0.97 [-2.19, 0.26]
Li,2008	46.75	6.42	6	53.82	3.78	6	10.2%	-1.24 [-2.52, 0.04]
**Total (95% CI)**			**91**			**54**	**100.0%**	**-2.20 [-3.15, -1.26]**

Heterogeneity: Tau² = 1.85; Chi² = 40.44, df = 11 (P < 0.0001); I² = 73%
Test for overall effect: Z = 4.57 (P < 0.00001)

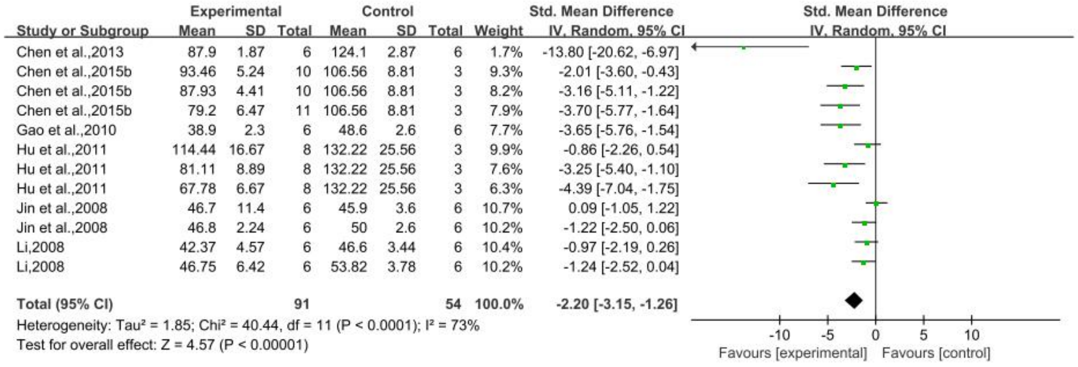

(A)

Study or Subgroup	Experimental Mean	Experimental SD	Experimental Total	Control Mean	Control SD	Control Total	Weight	Std. Mean Difference IV, Random, 95% CI
Jing et al.,2017	46.25	0.63	8	80.63	0.63	8	0.4%	-51.59 [-72.21, -30.98]
Shi,2017	31.22	9.54	9	31.11	10.72	9	18.1%	0.01 [-0.91, 0.93]
Tian et al.,2018	20.22	4.94	8	28.43	1.91	4	16.0%	-1.78 [-3.26, -0.30]
Tian et al.,2018	16.29	3.6	8	28.43	1.91	4	13.5%	-3.51 [-5.61, -1.42]
Zhu,2006	67.67	23.99	6	63.67	14.62	6	17.4%	0.19 [-0.95, 1.32]
Zhu,2006	65.17	14.8	6	73.5	17.4	6	17.3%	-0.48 [-1.63, 0.68]
Zhu,2006	96.33	42.93	6	102.67	39.32	6	17.4%	-0.14 [-1.28, 0.99]
**Total (95% CI)**			**51**			**43**	**100.0%**	**-1.05 [-2.41, 0.31]**

Heterogeneity: Tau² = 2.44; Chi² = 37.38, df = 6 (P < 0.00001); I² = 84%
Test for overall effect: Z = 1.51 (P = 0.13)

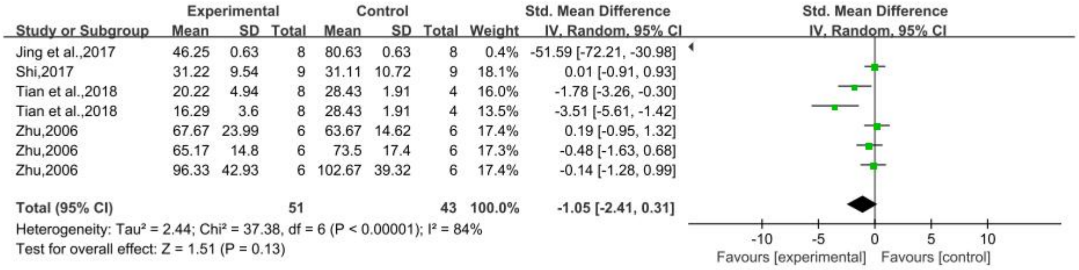

(B)

Study or Subgroup	Experimental Mean	Experimental SD	Experimental Total	Control Mean	Control SD	Control Total	Weight	Std. Mean Difference IV, Random, 95% CI
Hamzeh et al.,2014	114.92	8.66	6	152.05	12.99	3	5.2%	-3.27 [-5.72, -0.83]
Hamzeh et al.,2014	79.56	2.17	6	152.05	12.99	3	1.7%	-8.97 [-14.67, -3.27]
Li et al.,2017	110	21.9	6	134	19.2	2	6.8%	-0.97 [-2.71, 0.76]
Li et al.,2017	85	13.7	6	134	19.2	2	5.0%	-2.89 [-5.44, -0.34]
Li et al.,2017	74	10.1	6	134	19.2	2	3.6%	-4.31 [-7.68, -0.94]
Tan.,2003a	199.3	21.6	10	256.2	24	3	6.9%	-2.40 [-4.10, -0.70]
Tan.,2003a	237	23.1	10	256.2	24	3	7.8%	-0.77 [-2.11, 0.57]
Tan.,2003a	206.7	22.7	10	256.2	24	3	7.1%	-2.01 [-3.59, -0.42]
Tan.,2003a	235.4	20	10	256.2	24	3	7.7%	-0.93 [-2.29, 0.43]
Tan.,2003b	154.5	17.8	5	294.8	38.5	2	2.6%	-5.04 [-9.35, -0.72]
Tan.,2003b	190.3	28.9	5	260.2	15.4	2	5.3%	-2.20 [-4.59, 0.19]
Tan.,2003b	140.3	16.4	5	294.8	38.5	2	2.2%	-5.75 [-10.60, -0.91]
Tan.,2003b	175.2	20.2	5	294.8	38.5	2	3.3%	-4.04 [-7.63, -0.44]
Tan.,2003b	141.8	18.9	5	294.8	38.5	2	2.4%	-5.34 [-9.88, -0.80]
Tan.,2003b	174.4	13.3	5	294.8	38.5	2	2.7%	-4.84 [-9.02, -0.67]
Xue,2019	45.15	4.55	9	52.38	6.13	3	7.5%	-1.36 [-2.83, 0.11]
Xue,2019	38.25	6.4	10	52.38	6.13	3	7.1%	-2.07 [-3.67, -0.47]
Xue,2019	32.19	7.64	10	52.38	6.13	3	6.8%	-2.54 [-4.28, -0.80]
Yokozawa et al.,1997	42.43	2.65	9	39.78	5	8	8.6%	0.64 [-0.34, 1.62]
**Total (95% CI)**			**138**			**53**	**100.0%**	**-2.25 [-3.05, -1.44]**

Heterogeneity: Tau² = 1.70; Chi² = 49.17, df = 18 (P = 0.0001); I² = 63%
Test for overall effect: Z = 5.48 (P < 0.00001)

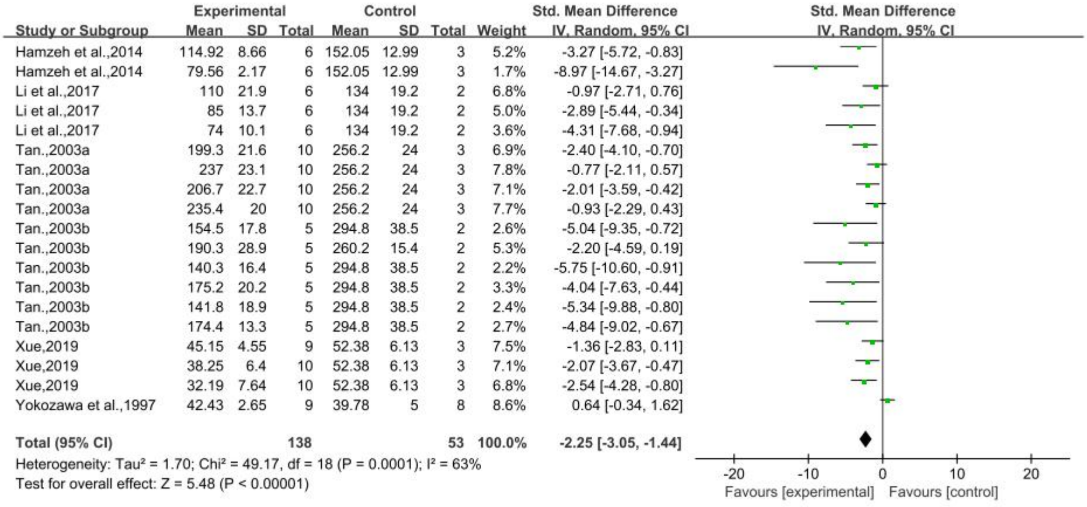

(C)

Figure S2 (A)Forest plot of Scr with rhein; (B)Forest plot of Scr with emodin; (C)Forest plot of Scr with rhubarb
