# Supplementary material for: The Effects of Rhubarb for the Treatment of Diabetic Nephropathy in Animals: A Systematic Review and Meta-analysis
Source: Front Pharmacol. 2021 Jun 11;12:602816. doi: 10.3389/fphar.2021.602816 (PMC8226322; doi:10.3389/fphar.2021.602816)
Supplement: Supplementary file 3 [file Image3.pdf]

Study or Subgroup	Experimental Mean	Experimental SD	Experimental Total	Control Mean	Control SD	Control Total	Weight	Std. Mean Difference IV, Random, 95% CI
Ai et al.,2004	8.39	1.13	9	11.36	1.79	8	23.6%	-1.91 [-3.11, -0.71]
Chen et al.,2013	11.21	4.27	6	19.12	4.32	6	17.3%	-1.70 [-3.10, -0.30]
Hu et al.,2011	7.68	1.95	8	17.56	3.54	3	6.1%	-3.77 [-6.14, -1.40]
Hu et al.,2011	13.29	1.71	8	17.56	3.54	3	13.2%	-1.74 [-3.34, -0.13]
Hu et al.,2011	6.1	1.34	8	17.56	3.54	3	3.8%	-5.12 [-8.11, -2.14]
Wang et al.,2012	9.12	1.65	8	12.22	1.62	3	13.3%	-1.72 [-3.33, -0.12]
Wang et al.,2012	8.46	1.55	9	12.22	1.62	3	11.8%	-2.22 [-3.92, -0.52]
Wang et al.,2012	8.12	1.42	10	12.22	1.62	3	10.9%	-2.62 [-4.38, -0.85]
**Total (95% CI)**			**66**			**32**	**100.0%**	**-2.18 [-2.76, -1.59]**

Heterogeneity: Tau² = 0.00; Chi² = 6.95, df = 7 (P = 0.43); I² = 0%
Test for overall effect: Z = 7.31 (P < 0.00001)

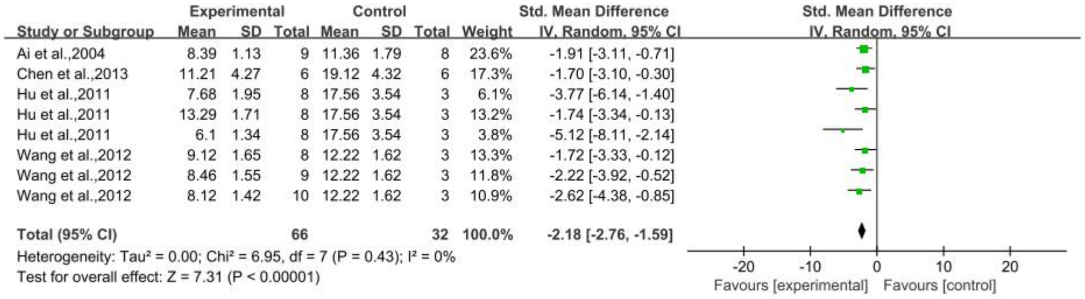

(A)

Study or Subgroup	Experimental Mean	Experimental SD	Experimental Total	Control Mean	Control SD	Control Total	Weight	Std. Mean Difference IV, Random, 95% CI
Shi,2017	7.06	1.47	9	7.81	1.44	9	21.2%	-0.49 [-1.43, 0.45]
Tian et al.,2018	10	3.2	8	13.54	2.92	4	15.0%	-1.05 [-2.35, 0.26]
Tian et al.,2018	8.15	1.85	8	13.54	2.92	4	11.2%	-2.24 [-3.86, -0.61]
Zhu,2006	12.77	4.11	6	11.43	2.19	6	17.4%	0.38 [-0.77, 1.52]
Zhu,2006	10.28	3.79	6	9.65	1.79	6	17.6%	0.20 [-0.94, 1.33]
Zhu,2006	15.92	12.22	6	18.1	10.86	6	17.6%	-0.17 [-1.31, 0.96]
**Total (95% CI)**			**43**			**35**	**100.0%**	**-0.44 [-1.09, 0.20]**

Heterogeneity: Tau² = 0.28; Chi² = 8.87, df = 5 (P = 0.11); I² = 44%
Test for overall effect: Z = 1.35 (P = 0.18)

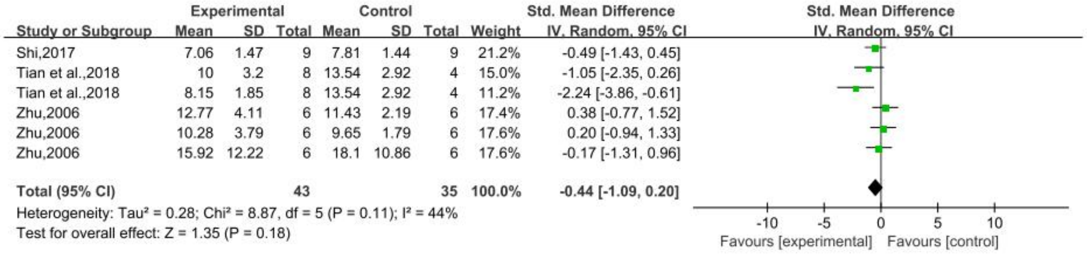

(B)

Study or Subgroup	Experimental Mean	Experimental SD	Experimental Total	Control Mean	Control SD	Control Total	Weight	Std. Mean Difference IV, Random, 95% CI
Hamzeh et al.,2014	14.04	2.08	6	25.81	0.15	3	2.5%	-5.95 [-9.86, -2.03]
Hamzeh et al.,2014	9.44	0.96	6	25.81	0.15	3	0.4%	-17.85 [-28.93, -6.76]
Li et al.,2017	7.6	1.5	6	12.5	1.5	2	4.3%	-2.84 [-5.37, -0.32]
Li et al.,2017	6.7	0.8	6	12.5	1.5	2	2.5%	-5.29 [-9.27, -1.32]
Li et al.,2017	10.2	2.1	6	12.5	1.5	2	5.9%	-0.99 [-2.73, 0.75]
Tan.,2003a	4.8	1.4	10	4.6	1.8	3	6.9%	0.13 [-1.17, 1.42]
Tan.,2003a	4.7	2.2	10	4.6	1.8	3	6.9%	0.04 [-1.25, 1.33]
Tan.,2003a	4.4	2.1	10	4.6	1.8	3	6.9%	-0.09 [-1.38, 1.20]
Tan.,2003a	4.6	1.9	10	4.6	1.8	3	6.9%	0.00 [-1.29, 1.29]
Tan.,2003b	8.9	3.4	5	12.5	3.6	2	5.8%	-0.88 [-2.66, 0.90]
Tan.,2003b	10	4.7	5	10.3	2.4	2	6.1%	-0.06 [-1.70, 1.58]
Tan.,2003b	12.8	3.3	5	12.5	3.6	2	6.1%	0.08 [-1.57, 1.72]
Tan.,2003b	10.2	5.4	5	10.3	2.4	2	6.1%	-0.02 [-1.66, 1.62]
Tan.,2003b	10.2	2.7	5	12.5	3.6	2	5.9%	-0.67 [-2.39, 1.06]
Tan.,2003b	11.9	4.5	5	10.3	2.4	2	6.0%	0.32 [-1.34, 1.98]
Xue,2019	10.31	2.05	9	17.09	2.59	3	5.5%	-2.89 [-4.81, -0.96]
Xue,2019	8.31	2.33	10	17.09	2.59	3	5.2%	-3.43 [-5.47, -1.39]
Xue,2019	5.15	1.04	10	17.09	2.59	3	2.7%	-7.66 [-11.41, -3.90]
Yokozawa et al.,1997	18.29	3.38	9	19.36	2.46	8	7.6%	-0.34 [-1.30, 0.62]
Yokozawa et al.,1997	0	0	0	0	0	0	0.0%	-0.34 [-1.30, 0.62]
**Total (95% CI)**			**138**			**53**	**100.0%**	**-1.18 [-1.92, -0.43]**

Heterogeneity: Tau² = 1.66; Chi² = 58.07, df = 18 (P < 0.00001); I² = 69%
Test for overall effect: Z = 3.10 (P = 0.002)

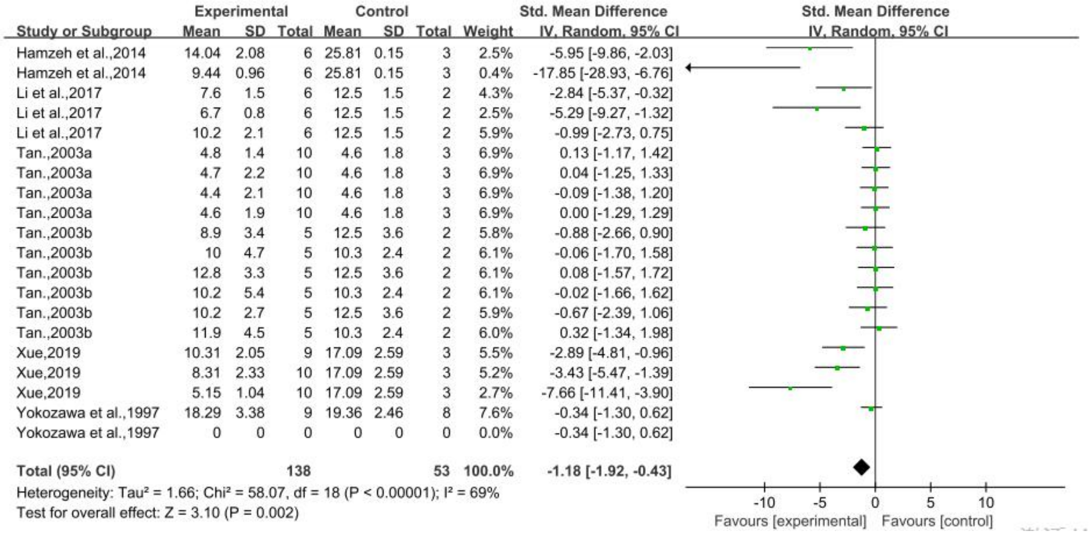

(C)

Figure S3 (A)Forest plot of BUN with rhein; (B)Forest plot of BUN with emodin; (C)Forest plot of BUN with rhubarb
