# Supplementary material for: The Effects of Rhubarb for the Treatment of Diabetic Nephropathy in Animals: A Systematic Review and Meta-analysis
Source: Front Pharmacol. 2021 Jun 11;12:602816. doi: 10.3389/fphar.2021.602816 (PMC8226322; doi:10.3389/fphar.2021.602816)
Supplement: Supplementary file 4 [file Image1.pdf]

Study or Subgroup	Experimental Mean	SD	Total	Control Mean	SD	Total	Weight	Std. Mean Difference IV, Random, 95% CI
Ai et al.,2004	29.72	2.28	9	30.57	2.56	8	8.8%	-0.33 [-1.30, 0.63]
Chen et al.,2013	24.03	1.24	6	26.12	7.89	6	7.9%	-0.34 [-1.49, 0.80]
Chen et al.,2015b	23.15	3.56	10	27.27	3.63	3	6.8%	-1.07 [-2.45, 0.31]
Chen et al.,2015b	19.14	1.55	11	27.27	3.63	3	4.4%	-3.71 [-5.78, -1.65]
Chen et al.,2015b	22.21	3.11	10	27.27	3.63	3	6.5%	-1.47 [-2.92, -0.01]
Chen,2015a	13.08	1.55	8	19.34	1.12	8	4.6%	-4.38 [-6.38, -2.37]
Gao et al.,2010	31.1	8.32	6	42.3	4.63	6	6.9%	-1.54 [-2.89, -0.18]
Hu et al.,2014	25.64	2.37	20	29.06	1.88	20	10.0%	-1.57 [-2.28, -0.85]
Li,2010	16.2	2.32	11	23.1	2.56	10	7.4%	-2.72 [-3.97, -1.47]
Liu and Ning,2007	19.6	1.4	20	21.3	2.3	20	10.4%	-0.88 [-1.53, -0.22]
Wang et al.,2012	18.65	1.8	8	19.8	1.86	3	6.9%	-0.58 [-1.94, 0.78]
Wang et al.,2012	18.32	1.81	9	19.8	1.86	3	6.9%	-0.75 [-2.11, 0.61]
Wang et al.,2012	18.12	1.74	10	19.8	1.86	3	6.9%	-0.89 [-2.24, 0.47]
Wang,2008	13.93	1.31	8	19.05	1.62	8	5.8%	-3.29 [-4.92, -1.65]
**Total (95% CI)**			**146**			**104**	**100.0%**	**-1.48 [-2.02, -0.94]**

Heterogeneity: Tau² = 0.63; Chi² = 35.98, df = 13 (P = 0.0006); I² = 64%
Test for overall effect: Z = 5.37 (P < 0.00001)

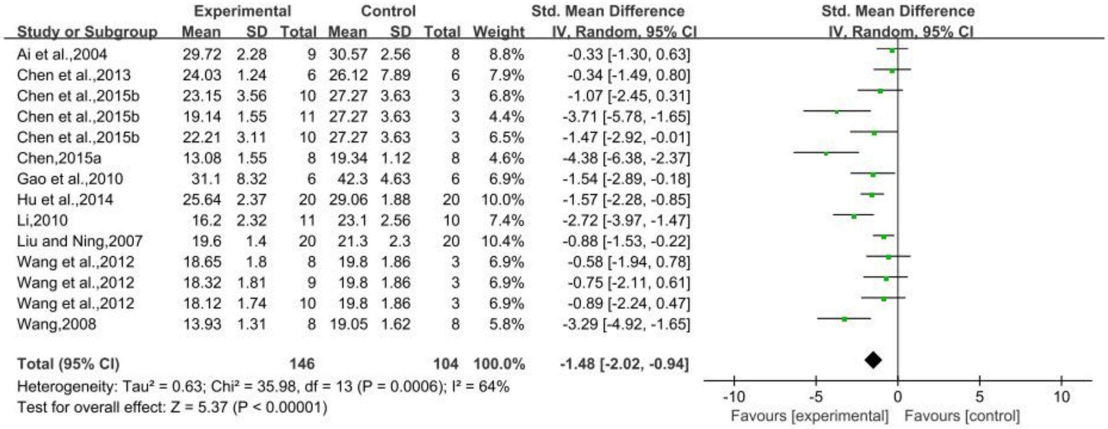

(A)

Study or Subgroup	Experimental Mean	SD	Total	Control Mean	SD	Total	Weight	Std. Mean Difference IV, Random, 95% CI
Chen et al.,2015c	27.44	1.41	12	28.59	1.28	12	15.5%	-0.82 [-1.66, 0.02]
Gao et al.,2014	20.21	0.83	4	21.25	1.25	4	10.9%	-0.85 [-2.36, 0.65]
Jing et al.,2017	16.15	0.77	8	24.42	0.96	8	3.5%	-8.99 [-12.70, -5.27]
Shi,2017	24.55	1.12	9	25.43	1.03	9	14.6%	-0.78 [-1.75, 0.19]
Tian et al.,2018	0	0	0	0	0	0		Not estimable
Yang et al.,2016	32.39	2.78	12	33.11	3.02	12	15.7%	-0.24 [-1.04, 0.56]
Zhu,2006	31.37	1.29	6	31.13	2.8	6	13.4%	0.10 [-1.03, 1.23]
Zhu,2006	29.33	3.09	6	31.37	2.67	6	13.1%	-0.65 [-1.83, 0.52]
Zhu,2006	27.3	4.58	6	25.32	6.88	6	13.3%	0.31 [-0.83, 1.45]
**Total (95% CI)**			**63**			**63**	**100.0%**	**-0.71 [-1.48, 0.05]**

Heterogeneity: Tau² = 0.80; Chi² = 24.61, df = 7 (P = 0.0009); I² = 72%
Test for overall effect: Z = 1.83 (P = 0.07)

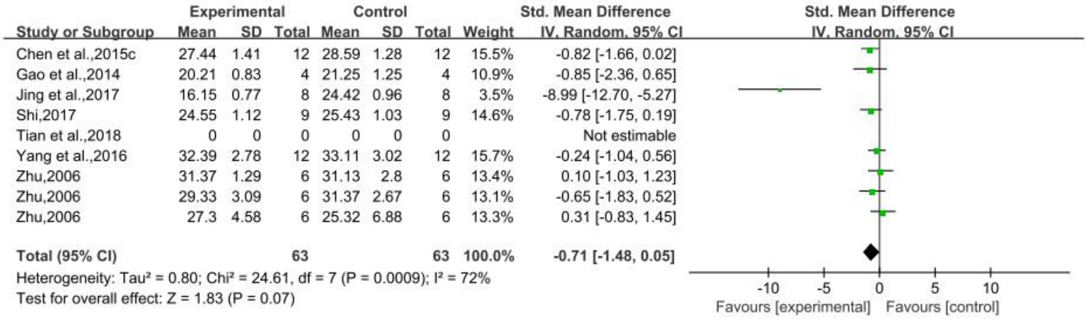

(B)

Study or Subgroup	Experimental Mean	SD	Total	Control Mean	SD	Total	Weight	Std. Mean Difference IV, Random, 95% CI
Hamzeh et al.,2014	6.59	0.19	6	20.65	0.93	3	0.1%	-23.92 [-38.73, -9.12]
Hamzeh et al.,2014	10.93	0.6	6	20.65	0.93	3	0.5%	-12.17 [-19.79, -4.54]
Li et al.,2017	22.1	2.8	6	25.7	5.1	2	4.7%	-0.95 [-2.68, 0.78]
Li et al.,2017	21.8	2.3	6	25.7	5.1	2	4.5%	-1.15 [-2.93, 0.64]
Li et al.,2017	24.4	3.4	6	25.7	5.1	2	4.9%	-0.30 [-1.92, 1.31]
Ni,2004	28.4	1.3	6	39	6.3	6	5.1%	-2.15 [-3.69, -0.61]
Ni,2004	29.3	5.1	6	29.7	7.3	6	6.1%	-0.06 [-1.19, 1.07]
Ni,2004	33.2	5.3	6	38.2	8.7	6	6.0%	-0.64 [-1.81, 0.53]
Tan.,2003a	20.2	7.1	10	22.2	6.4	3	5.7%	-0.27 [-1.56, 1.03]
Tan.,2003a	22.4	8.8	10	22.2	6.4	3	5.7%	0.02 [-1.27, 1.31]
Tan.,2003a	21.5	8.6	10	22.2	6.4	3	5.7%	-0.08 [-1.37, 1.21]
Tan.,2003a	21.9	7.5	10	22.2	6.4	3	5.7%	-0.04 [-1.33, 1.25]
Tan.,2003b	22.4	4.8	5	25.1	5.5	2	4.8%	-0.46 [-2.14, 1.22]
Tan.,2003b	22.2	4.2	5	25.1	5.5	2	4.7%	-0.54 [-2.24, 1.15]
Tan.,2003b	17.5	3.1	5	17.4	2.6	2	4.9%	0.03 [-1.61, 1.67]
Tan.,2003b	17	3.1	5	17.4	2.6	2	4.9%	-0.11 [-1.75, 1.53]
Tan.,2003b	17.7	4.8	5	17.4	2.6	2	4.9%	0.06 [-1.58, 1.70]
Tan.,2003b	21.1	3.8	5	25.1	5.5	2	4.6%	-0.80 [-2.56, 0.96]
Xue,2019	15.19	3.15	9	31.27	6.32	3	3.6%	-3.72 [-5.96, -1.48]
Xue,2019	11.4	2.44	10	31.27	6.32	3	2.8%	-5.31 [-8.07, -2.54]
Xue,2019	8.29	4.91	10	31.27	6.32	3	3.5%	-4.11 [-6.41, -1.82]
Yokozawa et al.,1997	26.46	2.68	9	30.17	4.93	8	6.4%	-0.90 [-1.92, 0.11]
**Total (95% CI)**			**156**			**71**	**100.0%**	**-0.96 [-1.53, -0.38]**

Heterogeneity: Tau² = 1.06; Chi² = 55.41, df = 21 (P < 0.0001); I² = 62%
Test for overall effect: Z = 3.27 (P = 0.001)

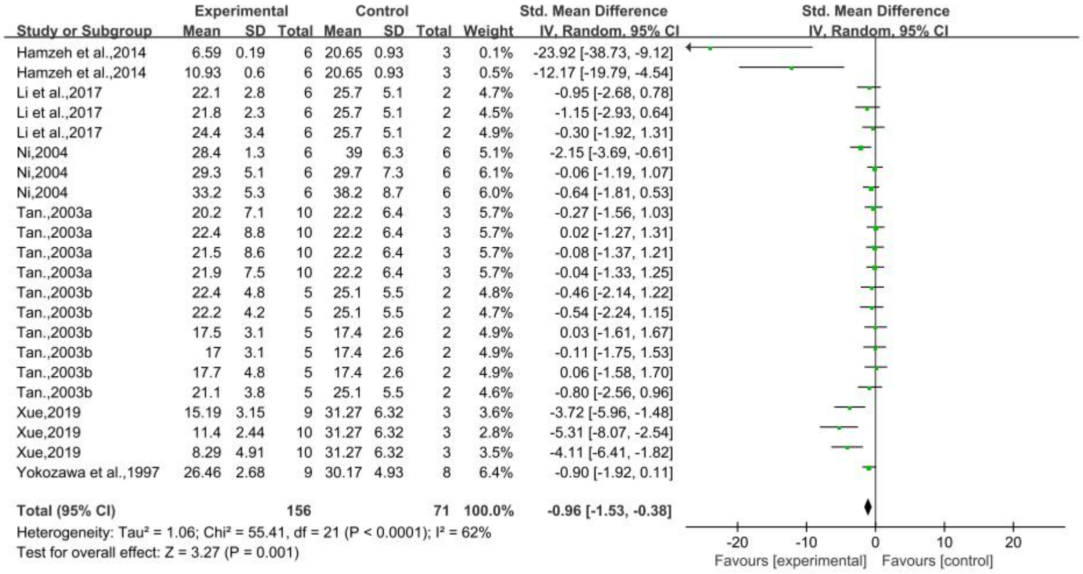

(C)

Figure S1 (A)Forest plot of blood glucose with rhein; (B)Forest plot of blood glucose with emodin; (C)Forest plot of blood glucose with rhubarb
